# Tracing Hepatitis E Virus in Pigs From Birth to Slaughter

**DOI:** 10.3389/fvets.2019.00050

**Published:** 2019-02-27

**Authors:** Jesper S. Krog, Lars E. Larsen, Solvej Ø. Breum

**Affiliations:** National Veterinary Institute, Technical University of Denmark, Kongens Lyngby, Denmark

**Keywords:** hepatitis E virus, zoonotic transmission, HEV, swine, infection dynamic, liver

## Abstract

Pigs are considered the main reservoir of genotypes 3 and 4 of the human pathogen hepatitis E virus (HEV). These viruses are prevalent at a high level in swine herds globally, meaning that consumers may be exposed to HEV from the food chain if the virus is present in pigs at slaughter. The aim of this study was to determine the HEV infection dynamics from birth to slaughter using 104 pigs from 11 sows in a single production system. Serum was collected from sows at 2 weeks prior to farrowing, in addition feces and serum samples were collected from the pigs every second week, from week 1 to week 17. Feces and selected organs were also sampled from 10 pigs following slaughter at week 20. All the samples were tested for HEV RNA by real-time RT-PCR and the serum samples were tested for HEV-specific antibodies using a commercial ELISA. Maternal antibodies (MAbs) were only present in pigs from sows with high levels of antibodies and all pigs, except one, seroconverted to HEV during weeks 13–17. In total, 65.5% of the pigs tested positive for HEV RNA at least once during the study (during weeks 13, 15, and/or 17) and significantly fewer pigs with a high level of MAbs became shedders. In contrast, the level of MAbs had no impact on the time of onset and duration of virus shedding. HEV was detected in feces and organs, but not in muscle, in 3 out of 10 pigs at slaughter, indicating that detection of HEV in feces is indicative of an HEV positivity in organs. In conclusion, a high proportion of pigs in a HEV positive herd were infected and shed virus during the finisher stage and some of the pigs also contained HEV RNA in feces and organs at slaughter. The presence of MAbs reduced the prevalence of HEV shedding animals, therefore, sow vaccination may be an option to decrease the prevalence of HEV positive animals at slaughter.

## Introduction

Hepatitis E virus (HEV) can cause severe infections in humans. Four genotypes of HEV are known; genotypes 1 and 2 are exclusively found in humans whereas genotypes 3 and 4 have been found in humans and pigs. Genotype 3 is found worldwide in pigs and in humans, while genotype 4 has mainly been found in both pigs and humans in Asia, and only more recently also in Europe (1). In several European countries, there has been a dramatic increase in human cases of HEV infection caused by Genotype 3 strains. These viruses have a high sequence identity to contemporary strains circulating in pigs, indicating that swine-to-human transmission of HEV is a common event (2). Indeed, high prevalence of anti-HEV antibodies (Abs) in swine herds has been reported from several countries. Detection of the high HEV seroprevalence in older samples indicated that HEV has been present in pigs for decades. A number of studies have shown that consumers are indeed exposed to HEV since porcine livers, bought in supermarkets, have been found to contain HEV-specific RNA (3–5). Furthermore, HEV, isolated from commercial livers, has been shown to be infectious for pigs in an experimental trial (3). In another study, a pig liver sausage, Figatellu, which is traditionally eaten raw, was found to be the cause of hepatitis in a significant number of people who consumed it (6). In addition, 2–15% of pigs have been shown to be infected with HEV at slaughter (7). Previous longitudinal studies, performed in pigs, revealed that most of the pigs became infected at 8–15 weeks of age but some of the pigs were still positive at slaughter (8–10). Maternal antibodies (MAbs) against HEV have been shown to be successfully transferred from HEV-Ab positive sows to offspring. However, in a previous study comparing a few animals in a single herd, the level of MAbs had no impact on the infection dynamic of HEV in the offspring. Thus, the protective role of MAbs in pigs is presently unclear (11). The proven zoonotic potential of HEV in pigs combined with the relatively high prevalence of HEV positive pigs in Denmark (more than 50% of the sow herds are HEV positive) (12) may have a negative impact on the safety of Danish pork products if the virus is present in Danish pigs at slaughter. Thus, it is essential to obtain a better knowledge of HEV infection dynamics in typical pig production systems. The aim of the present study was to study the HEV infection dynamics from birth to slaughter, with special focus on the impact of maternal antibody levels and the infectious status of individual pigs at slaughter. Furthermore, the distribution of HEV in different tissues of naturally infected pigs that shed virus 3 weeks prior to slaughter was examined.

## Materials and Methods

### Field Study Design and the Study Herd

A longitudinal study was performed in a single farrow-to finisher herd. More than 100 crossbred pigs were sampled every second week from birth to slaughter. The pigs were kept at the breeding unit until they reached ~30 kg after which they were moved to the finisher site situated ~16 km from the breeding unit. Before initiating the study, the presence of HEV in the first parity sows (gilts) at the nursery site was determined by testing feces from 10 sows using an HEV specific real time RT-PCR assay (data not shown).

### Selection of Sows

Two weeks prior to farrowing, serum samples were collected from 58 sows and tested for HEV Abs. Based on the measured, normalized levels of HEV Abs, the sows were divided into three groups; low (1 ≤ OD < 2), intermediate (2 ≤ OD < 3) and high (OD≥ 3) levels of HEV-specific Abs. The group of low level HEV Abs comprised 23 sows with a mean normalized OD of 1.38 (SD = 0.27). The groups with intermediate and high levels of HEV Abs each included 17 sows, with mean normalized OD values of 2.44 (SD = 0.24) and 4.50 (SD = 1.47), respectively. The farmer randomly selected four sows from each group to be included in the study. Just after farrowing, all piglets from the 12 sows were ear tagged with a unique number. If more than half of the piglets within a litter died, the sow and her piglets were excluded from the study.

### Sampling of Pigs

One week after farrowing, blood sampling of all piglets was performed by a local pig health technician. Thereafter, both rectal swabs and blood samples were collected every second week until week 17 from all piglets. The pigs were restrained either manually or with a snout break and 9 mL of blood was collected by puncture of the jugular vein. The rectal swabs were collected, using a cotton swab, at the rectal surface ~2–3 cm from the anus and then placed into a sterile container with 2 mL PBS. The samples were labeled and kept cool during transportation to the laboratory. The blood samples were stored at 4°C until further processing on the same day. The serum was extracted from whole blood by centrifugation at 3,000 RPM for 10 min at 5°C. The serum fractions were then transferred into Nunc tubes and stored at −80°C until RNA extraction. The tubes containing the cotton swabs in 2 mL PBS were shaken at 300 rpm for 1 h before the liquid was poured into 2 mL Eppendorf tubes and stored at −80°C until analysis. Individual pigs were excluded from the study if more than two sampling dates were missed.

### Selection of Pigs for Tissue Sampling

Ten of the 26 pigs where shedding of HEV (as detected by the presence of HEV RNA) occurred ~3 weeks prior to slaughter (week 17), were randomly selected for necropsy at a laboratory facility situated 100 km from the herd. At the age of 20 weeks, the pigs were transported alive to the laboratory on a vehicle with no other pigs present. On arrival, the pigs were killed by intra-cardiac injection of pentobarbiturate (50 mg/kg) and exsanguinated by cutting the arteria axillaris. At necropsy, samples of the tonsils, lungs, kidneys, spinal cord, gall bladder (intact), hepatic lymph nodes, colon with contents, small intestine with contents, mesenteric lymph nodes, heart, and the entire liver were collected. Furthermore, muscle samples (3 × 3 cm) were collected from the shoulder, neck, pork loin, tenderloin, ham, and diaphragm. Intestinal contents were collected from the colon and the small intestine. The tissue was then rinsed in cold PBS. Bile was extracted from the gall bladder with a syringe and a small piece of tissue was excised and rinsed in PBS to remove the remaining bile. All samples were transferred to labeled tubes and stored at −80°C until analysis.

### RNA Extraction and PCR Analysis

Automated extraction of RNA from the rectal swab supernatant was performed on the QIAsymphony SP system (QIAGEN) using the DSP virus/pathogen mini kit version 1 (QIAGEN, Cat no. 937036). The protocol used was complex 200 V5 DSP with an elution volume of 110 μL. The HEV RNA was detected by real time RT-PCR essentially as described by Breum et al. (12) except that the concentration of the primers was changed to 500 nM for HEV2-P and HEV2-R and 100 nM for HEV2-F. Furthermore, the time settings used for the PCR cycling were changed to 15 s for denaturation and annealing and 20 s for elongation.

### Serological Analysis

All serum samples were tested for the presence of anti-HEV IgG using a commercial kit (PrioCHECK® HEV Ab porcine kit; Prionics). As recommended by the vendor, only the samples having an OD value that exceeded the OD of the cut-off control (provided in the kit) multiplied by 1.2 were regarded as positive. The OD values were normalized by dividing the OD of the sample with the OD of the cut-off control multiplied by 1.2, which eliminated plate-to-plate variations. According to the information provided by the vendor, the assay has a sensitivity of 91% and a specificity of 94%.

### Statistical Analysis

The statistical analyses were performed using SAS 9.1. For the determination of the overall difference between the three groups, a mixed linear model was used. This method allowed for missing data points from individual pigs. To evaluate the differences on a weekly basis, the ANOVA was performed. Finally, to compare groups for the difference in the number of shedders, the χ^2^-test was applied. For all analyses the significance level was set at *P* = 0.05.

## Results

Initially, a total of 12 sows and 135 piglets were included in the study, but 31 of the piglets, including one entire litter, either died or were excluded due to missing sampling points. Thus, data from a total of 104 piglets from eleven sows were included in the analysis.

### Serology

Based on the levels of HEV Abs prior to farrowing, the 11 sows were allocated to one of three groups with low, intermediate or high levels of HEV Ab, designated group 1, 2, and 3, respectively. Normalized OD values, indicative of the HEV Ab levels in serum, for the included sows and the number of piglets in each litter in each group are listed in Table 1.

**Table 1:** Grouping of piglets according to levels of HEV antibodies in sows prior to farrowing.

**Group**	**Group 1 (Low level of HEV IgG)**				**Group 2 (Intermediate level of HEV IgG)**			**Group 3 (High level of HEV IgG)**			
Sow ID	3399	3545	3485	3681	3266	3699	3548	3552	3532	3292	3145
OD (norm.)	1.3	1.4	1.4	1.4	2.5	2.8	2.9	5.8	6.1	6.9	11.6
# Piglets (in study/born)	8/8	15/19	6/10	9/10	10/11	8/10	15/18	8/10	9/10	8/10	8/10
Total # pigs	38				33			33			

**Figure 1:**
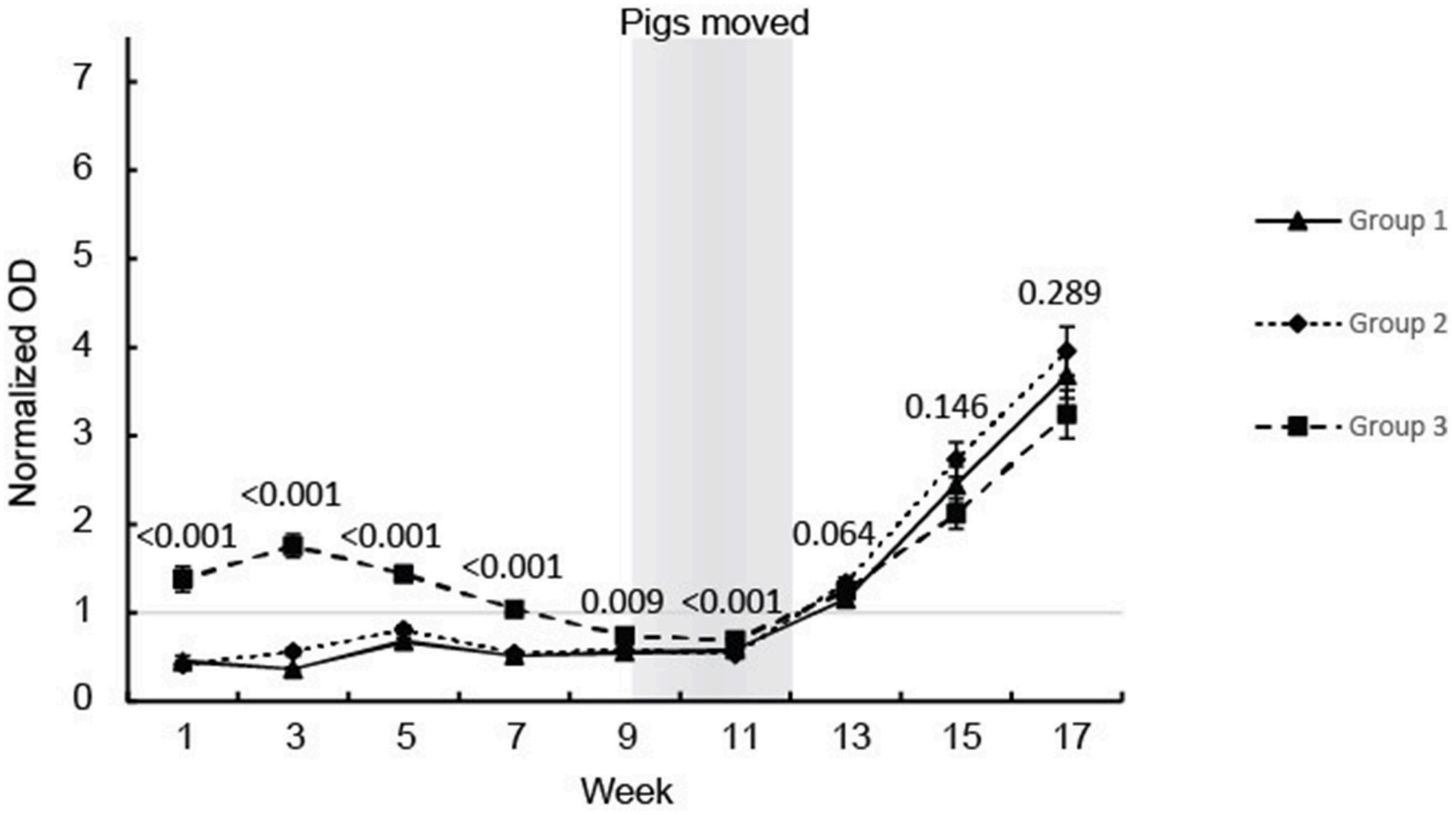
HEV antibody (IgG) development in the eartagged pigs. The values are expressed as mean values of the normalized ODs for the serum from pigs in the three groups. The results of the statistical analysis of the differences between the pigs in group 3 compared with groups 1 and 2 (ANOVA) are indicated at each sampling point.

All the pigs, except for one, seroconverted during the study (Figure 1). The pigs in groups 1 and 2 showed similar anti-HEV Ab profiles in serum with OD values below the cut off until seroconversion that occurred between weeks 11 and 13 followed by a steady further increase in HEV IgG levels which lasted until the end of the observation period at week 17 (Figure 1). Group 3 showed a different profile with positive HEV IgG levels from birth until week 7 and then these group 3 pigs, like the pigs in groups 1 and 2, seroconverted between week 11 and 13 followed by a steady increase in HEV IgG levels until week 17 (Figure 1). No differences were seen between the pigs in groups 1 and 2 so these groups were combined in the statistical analyses. There was a clear difference in the level of HEV IgG between the pigs in group 3 compared to the pigs in group 1 and 2 from week 1 to 11, but not at week 13 to 17 (Figure 1).

### Real Time RT-PCR

**Figure 2:**
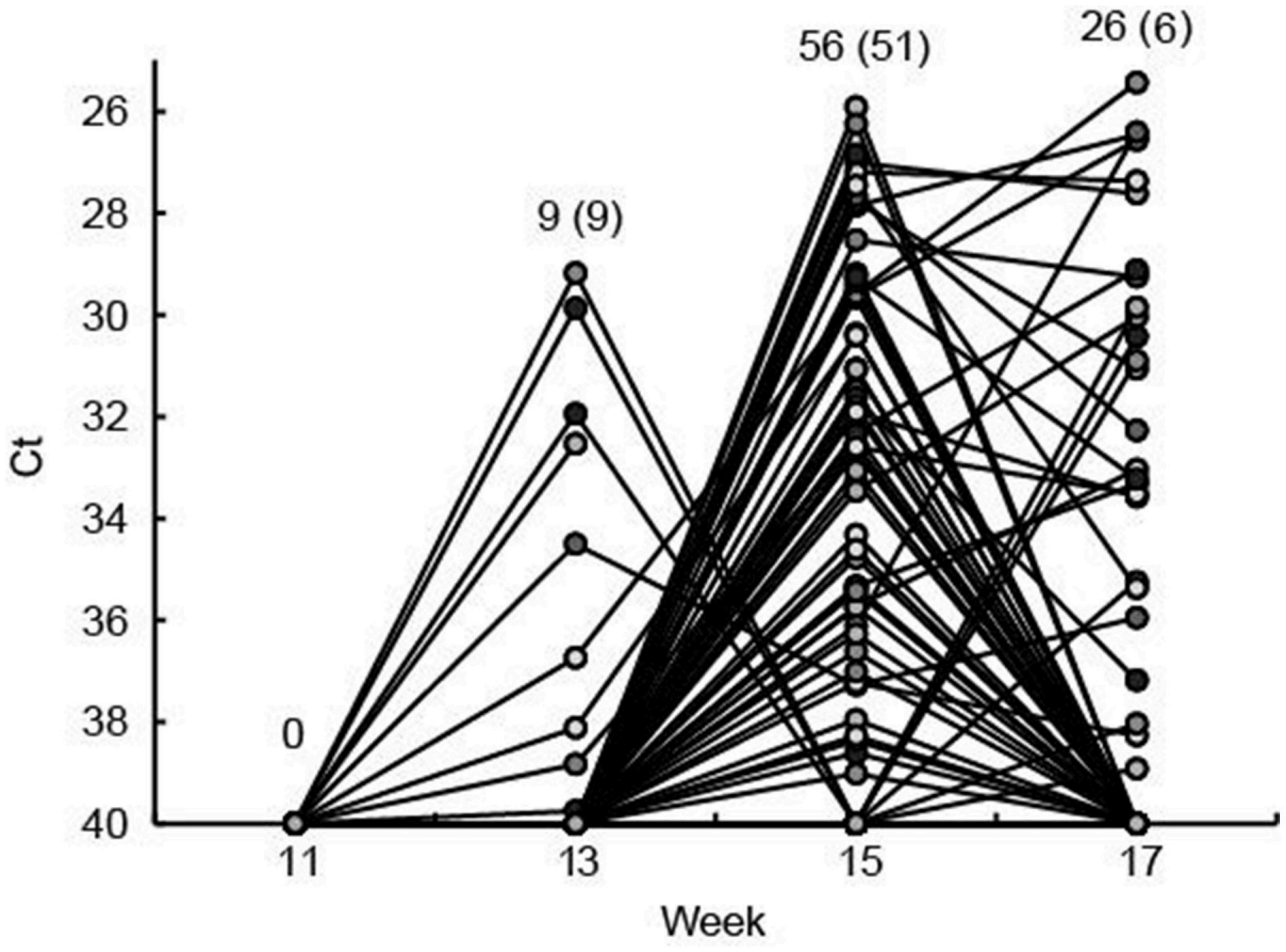
The fecal shedding of HEV from all eartagged pigs is shown as the Ct values obtained by real time RT-PCR testing of feces. The Ct scale has been inverted and negative samples have been set at Ct 40. Plain numbers indicate the total number of pigs positive for HEV RNA at 11, 13, 15, and 17 weeks of age and the numbers in parenthesis indicate the number of pigs that tested positive for the first time in that week. Each of the eartagged pigs are marked with a circle filled with different shades of gray.

Of the 104 ear marked pigs included in the analysis, 66 pigs (63.5%) tested positive for HEV RNA in feces in at least one sample during the study period (Table 2). There was a significant difference in the number of viral shedders ranging from ~73% in groups 1 and 2 to 45% for group 3 (*P* = 0.032) (Table 2). However, there was no significant difference in the time when the first detection of HEV shedding was observed between the groups (*P* = 0.876). None of the pigs tested positive for HEV prior to week 13 and only 9 pigs became virus positive between weeks 11 and 13 (Figure 2). The majority of the pigs (*n* = 51) tested positive for HEV for the first time at week 15, whereas six pigs tested positive for the first time at week 17. Of the 104 pigs, 23 (22%) tested positive for HEV in feces at two samplings and two pigs (2%) were positive at three samplings (weeks 13, 15, and 17) (Figure 2).

**Table 2:** The number of pigs that tested positive, for the first time, in each of the three groups.

	**Group 1 (low)**	**Group 2 (intermediate)**	**Group 3 (high)**	**Total**
Week 13	2	6	1	9
Week 15	21	17	13	51
Week 17	4	1	1	6
Total shedders	27/38 (73.7%)	24/33 (72.7%)	15/33 (45.5%)	66/104 (63.5%)

*Each individual pig is only included in the week when it tested positive for the first time*.

### Analysis of Samples Collected From Selected Pigs at Slaughter

**Figure 3:**
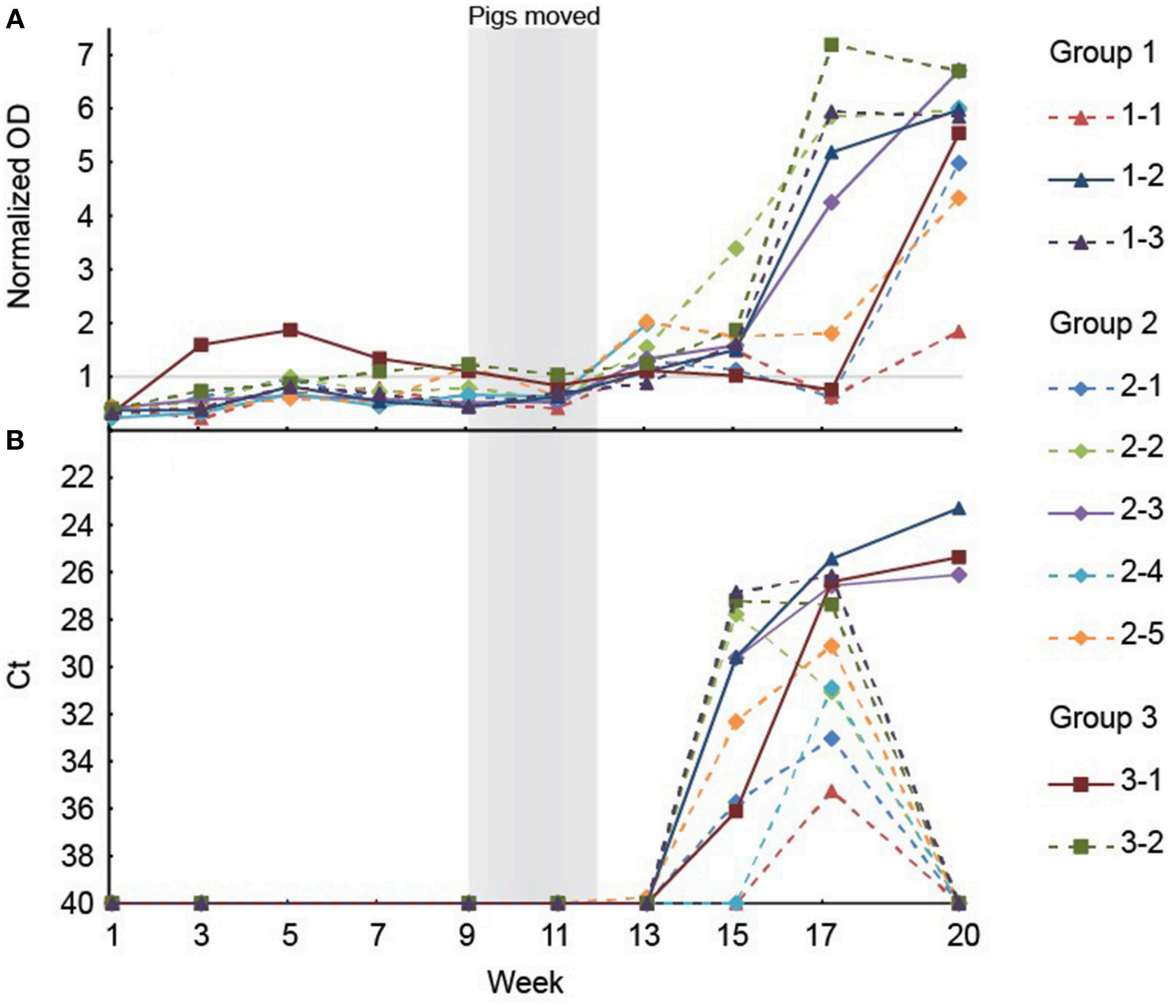
**(A)** The results of the anti-HEV IgG measurements in serum from the ten pigs selected for necropsy. Note that the serological data are missing for week 15 and 17 for the pig with ID 2–4. **(B)** Positive HEV tests of feces of the ten pigs selected for necropsy. The Ct scale has been inverted and negative samples have been set at 40 Ct.

To analyze if the organs and tissues contained HEV at slaughter, 10 of the 26 pigs that tested positive for HEV at week 17, ~3 weeks prior to slaughter, were randomly selected for further analysis. The 10 pigs included three, five and two pigs from groups 1, 2, and 3, respectively. The HEV IgG profiles for the 10 individual pigs from birth until slaughter are shown in Figure 3A. Three of the pigs (1-1, 2-1, and 3-1, one from each group denoted by the first number in the ID) were seronegative at week 17, but both pigs 2-1 and 3-1 had tested positive for HEV before week 15 (Figure 3B). At slaughter (week 20), three of the 10 pigs, one from each group, were still positive for HEV RNA in feces at a level similar to that observed at week 17 (Figure 3B). There was no significant difference in the HEV shedding pattern before week 17 for the three pigs that were positive for HEV at week 20 compared to the other seven pigs that tested negative for HEV at week 20 (*P* = 0.633). Interestingly, only the three pigs that tested positive for HEV in feces at week 20 were positive for HEV RNA in organs (Table 3). Only the internal organs tested positive for HEV RNA while none of the muscle samples tested positive. The liver associated samples [liver, bile, gall bladder, and hepatic lymph nodes (HLN)] were strongly positive for HEV RNA (low Ct) whereas lower levels of HEV RNA were detected in extra-hepatic organs such as the lungs and tonsils.

**Table 3:** Detection of HEV RNA in samples collected from necropsied pigs.

	**Pig 1–2**	**Pig 2–3**	**Pig 3–1**
	**C_**t**_**	**C_**t**_**	**C_**t**_**
Feces	23.3	26.11	25.4
Small intestine (contents)	27.0	27.9	-
Colon	–	–	–
Small intestine	–	–	–
Intestinal lymph node	–	37.2	38.3
Gall bladder	31.3	31.1	29.2
Bile	23.4	24.9	27.7
Liver	21.5	30.8	27.5
Hepatic lymph node	30.7	26.9	36.6
Kidney	–	–	–
Lung	34.7	34.1	35.3
Tonsil	–	38.8	38.3
Spinal cord	–	–	–
Muscle^*^	–	–	–
Heart	–	–	–

*Only the three pigs with positive samples are shown*.

* *Muscle included six different samples of muscle collected from parts of the pig used for food products. All samples were analyzed separately*.

## Discussion

The offspring from 11 sows with different levels of HEV specific antibodies were included in the present study. To investigate the efficacy of passive transfer of maternal antibodies on the HEV infection dynamic in the offspring, the 104 piglets were allocated to one of three groups based on the level of anti-HEV antibodies measured in the sows 2 weeks prior to farrowing. The MAbs were detected only in piglets from sows with high levels of anti-HEV Abs prior to farrowing, revealing a clear correlation between the levels of anti-HEV Abs in the sows and the maternal anti-HEV Abs in the piglets. This finding is in accordance with previous studies, which also showed that a high level of antibody is required for effective transfer from the sow (8–10). The difference in HEV MAbs levels between piglets born of sows with high level of HEV IgG (group 3) compared to the other two groups were significantly different until week 13. Previous studies have confirmed that MAbs against HEV decline at around weeks 9–13 (8, 9).

HEV RNA was detected in feces of pigs from week 13 and onwards. Thus, no viral shedding was detected in the pigs when housed in the sow herd because the pigs were moved to the finisher site at 30 kg (week 9–12). Based on the facts that anti-HEV Abs were detected in the sows prior to farrowing and that HEV RNA was detected in the gilts in the herd (data not shown), HEV was indeed present in the sow herd of this study. However, it is not clear, if the piglets were infected by HEV just prior to being moved from the breeding unit or if the pigs were infected after arrival at the finisher site. However, although there was no effect of the level of HEV MAbs on the onset or duration of viral shedding, significantly fewer pigs in the group with initially higher levels of MAbs tested positive for HEV during the study. These findings indicated that the pigs were exposed to HEV relatively late in the nursery period i.e., after the MAbs had declined in most pigs. A previous field study failed to show any effect on the level of MAbs on the risk of becoming HEV shedders, however, that study was performed on very few animals (2 litters) and the pigs were infected very early (week 3–4) indicating a high viral load in the environment (11). Another field study detected HEV RNA in feces of pigs starting in weeks 12–15 ~3–5 weeks after the anti-HEV MAbs had waned, which is more in line with the findings in the present study (9).

Seroconversion against HEV, as measured using a commercial HEV ELISA, was observed in the present study in all pigs, except one, starting between week 11 and week 13 which is in accordance with development of IgG in previous studies (8–10). Thus, the pigs that were HEV RNA negative at all samplings in feces also seroconverted indicating that they indeed were infected or at least exposed to HEV either in a short period of time or at levels below the detection limit of the real-time RT-PCR assay. However, these animals may have been positive for HEV in other tissues or in serum. Seroconversion coincided with the first detection of viral RNA for most of the pigs. This was unexpected since IgG Abs previously have been shown to develop 2–3 weeks after onset of viremia (9, 11). Detection of HEV in serum, in the present study, was attempted on the same sampling days as for the feces samples, but was unsuccessful even though different methods for RNA extraction were tested and the assay previously has performed very well in detecting HEV RNA in serum samples from the field (12) and in a ring trial (unpublished results). The level of HEV in serum has, however, previously been shown to be significantly lower than in feces and the viremia also seems to be of shorter duration than the fecal shedding (11). Furthermore, in an experimental trial in pigs, using intravenous inoculation of homogenates of livers with different levels of HEV, it was shown that the duration and levels of viremia were strongly correlated to the level of HEV present in the inoculum (3). Thus, a likely explanation for the finding in the present study, i.e., seroconversion coincided with positive fecal samples, could be that virus fecal excretion start days or even weeks after exposure. Another contributing factor to the early detection of anti-HEV Abs could be that the anti-porcine IgG conjugate included in the ELISA cross-reacted with IgM Abs which normally develop earlier than IgG (8, 10).

The HEV RNA was detected in internal organ samples (intestine, lymphatic tissue, bile and liver), but not in muscle, which is in accordance with previous findings (7, 11, 13, 14). Interestingly, only the pigs that tested positive in fecal samples at slaughter were also positive in organs. This indicated that testing of feces from pigs prior to slaughter could be used as an indicator of HEV presence in internal organs. However, albeit that all feces positive pigs were found to harbor HEV in tissue in one previously study (14), the predictive value of a negative feces test may be limited since HEV has been detected previously in organs from pigs that tested negative in feces (8, 14).

In conclusion, a high proportion of the pigs, in a single HEV positive herd, were infected and tested positive for HEV during the finisher stage and a fraction of these pigs also had HEV RNA in feces and organs at slaughter. High levels of MAbs reduced the prevalence of HEV positive animals and, therefore, sow vaccination may be an option to decrease the prevalence of HEV positive animals at slaughter, however, more studies are required to investigate this.

## Data Availability

The datasets generated for this study are available on request to the corresponding author.

## Ethics Statement

The study included blood sampling of animals under field conditions for diagnostic purposes and by that did not require approval from the ethic committee.

## Author Contributions

JK participated in the design of the study, performed the laboratory analyses on pig samples, participated in the assessment and statistical analysis and drafted the manuscript. LL participated in the design of the study and in the assessment and statistical analysis and commented and made adjustment to the manuscript. SB participated in the design of the study, in the establishment of the analytical assays and participated in the assessment and statistical analysis and commented and made adjustment to the manuscript.

### Conflict of Interest Statement

The authors declare that the research was conducted in the absence of any commercial or financial relationships that could be construed as a potential conflict of interest.
